# Prevalence of myopia and associated risk factors among primary students in Chongqing: multilevel modeling

**DOI:** 10.1186/s12886-020-01410-3

**Published:** 2020-04-15

**Authors:** Zhihao Xie, Yue Long, Jingxuan Wang, Qiaoqiao Li, Qiang Zhang

**Affiliations:** 1 Department of Epidemiology and Biostatistics, West China School of Public Health and West China Fourth Hospital, Sichuan University, #16, Section 3, Renmin Nan Lu, Chengdu, Sichuan 610041 People’s Republic of China; 2 Department of Ophthalmology, West China Hospital, Sichuan University, Chengdu, China; 3 School of Education, Soochow University, Suzhou, China

**Keywords:** Myopia, Prevalence, Risk factors, Playing electronics, Multilevel modeling

## Abstract

**Background:**

The prevalence of myopia and associated risk factors among children in Chongqing has not yet been determined. This study investigated the prevalence of myopia and possible relationships between myopia and several related factors among school children in Chongqing.

**Methods:**

This cross-sectional study assessed a sample of 997 children (7–13 years of age) attending primary school in Chongqing. Data were obtained from visual acuity and refractive error measurements and a structured questionnaire. Myopia was defined as visual acuity < 5.0 and refractive error (spherical equivalent) of < − 0.50 diopters (D) in either eye. Multilevel modeling was applied to investigate potential risk factors.

**Results:**

The overall prevalence of myopia was 33.9% [95% confidence interval (CI) = 31.0–36.8]; myopia prevalence significantly increased with age. Girls were at a higher risk of myopia than boys [odds ratio (OR) = 1.449, 95% CI = 1.060–1.979]. Children with paternal myopia (OR = 2.130, 95% CI = 1.376–3.297) or maternal myopia (OR = 1.861, 95% CI =1.153–3.002) had a higher risk of myopia than those without myopic parents. Children who spent more than 1 h daily outdoors were less likely to have myopia; meanwhile, children who did homework more than 3 h daily (OR = 2.237, 95% CI = 1.041–4.804), watched television more than 3 h daily (OR = 2.106, 95% CI = 1.200–3.697), or played electronics more than 1 h daily (OR = 2.983, 95% CI = 2.088–4.262) had a higher risk of myopia.

**Conclusions:**

Myopia in school children is a serious public health problem in Chongqing. Myopia was significantly positively associated with higher age, female sex, parental myopia, and spending a long time indoors; notably, playing with electronics had the greatest influence on the risk of myopia.

## Background

Myopia, which threatens visual quality and eye health, is a common refractive error among children and teenagers worldwide. Notably, the myopia prevalence is particularly high in east Asian countries; the prevalence in children 6–12 years of age is 53.9% in Tianjin, China [1], 36.7% in children aged 5–14 years in Beijing [2], 69.5% in teenage children in Singapore [3], 24.8% in 12-years-olds in Malaysia [4], 22.6% in Korean children [5]. Studies have suggested that both genetic and environmental factors have a marked impact on myopia in school children. Myopia risk factors thus far include parental myopia [6], near work activities [7, 8], spending a long time studying [2], and prolonged indoor activity [9]; meanwhile, extended outdoor activity has been associated with reduction in the incidence rate of myopia [10].

Notably, ordinary logistic regression was performed in many prior studies [1, 9, 11, 12] to identify myopia risk factors, although the data emanating from student environments are often hierarchically structured; moreover, the ordinary logistic regression model cannot recognize the existence of clustering, resulting in the underestimation of standard errors of regression coefficients [13]. A preferable statistical approach, multilevel modeling, is more suitable for analyzing these data, as it can identify the existence of clustering within hierarchically structured data, thereby improving estimation of effects and corresponding standard errors [14].

While the myopia prevalence among children in large cities in China, including Tianjin, Beijing, and Guangzhou [1, 10, 15], has previously been reported, the prevalence of myopia and its associated risk factors among children in Chongqing has not yet been determined though the city has the largest population in China, with nearly 40% of its population living in rural areas. Moreover, multilevel modeling was only used in the study conducted in Guangzhou [15]. Therefore, in the present study, we conducted a cross-sectional survey to determine the prevalence of myopia among primary students in Chongqing and investigated potential myopia risk factors by multilevel modeling.

## Methods

### Study design and population

In 2018, we conducted a cross-sectional survey of the prevalence of myopia and its associated risk factors in children attending primary schools from grades 1 to 6 in Dianjiang county, Chongqing city, China. Two-stage stratified cluster sampling was used to select students for study inclusion. In the first stage, 4 primary schools were randomly selected, including 2 rural schools and 2 urban schools. In the second stage, 1 class was randomly selected from each grade of the selected schools. We informed the school principals and class teachers of the related content and procedures of the study. All children in the selected classes were invited to participate in the study, except those with eye diseases. Written informed consent forms were obtained from the children’s parents before the eye examinations and questionnaires were administered. The study protocol was approved by the Sichuan University, West China School of Public Health Institutional Review Board.

### Eye examination and questionnaire

Visual acuity (VA) was assessed without refractive correction in all children using a logarithmic VA chart with a 5-point recording at 5 m. Refractometry was performed in all children in a noncycloplegic state by autorefractometry (auto-refractor KR-1, Topcon, Tokyo, Japan). The spherical equivalent refraction (SE) was calculated as the spherical refractive error + 1/2 of the cylindrical refractive error.

The questionnaire items addressed potential risk factors such as demographic characteristics (age, sex, class, and grade level), a genetic factor (parental myopia status), and multiple environmental factors such as the number of times the children performed eye exercises daily, class recess location, daily sleep duration, and time spent outdoors daily. Also, the number of hours spent performing indoor activities such as, doing homework, using computer, watching television and playing with electronics was assessed as well as whether weekly cramming study sessions were attended. Near work activities such as reading while in a dark environment, reading while lying down, and average reading distance.

### Definition of myopia

In our study, myopia was defined as VA (logMAR) < 5.0 and refractive error (spherical equivalent) of < − 0.50 diopters (D) in either eye [16]; these thresholds were applied to reduce the number of false-positive myopia results. Myopia was further divided into three refractive error groups: low myopia (< − 0.5D to ≥ − 3.0D), moderate myopia (< − 3.0D to ≥ − 6.0), and high myopia (< − 6.0D) [15].

### Statistical analysis

Statistical analysis was performed using R software (version 3.5.2; R Foundation for Statistical Computing, Vienna, Austria) and MLwiN software (version 3.03; Centre for Multilevel Modeling, University of Bristol, Bristol, UK). The chi-squared test was used to assess differences in myopia prevalence based on demographic parameters. The Cochran–Armitage test for trends was used to examine the association between myopia prevalence and grade level or age. The normality of the continuous variable distributions was assessed by the Shapiro–Wilk test. Data for continuous variables with normal distributions were presented as the mean ± standard deviation; data for non-normal variables were presented as the median ± interquartile range. Categorical variables were presented as frequencies of the total.

A complex, multi-stage sampling design was used and the students were grouped by classes; therefore, the variables may not be independent. Hence, a two-level multiple logistic regression was performed using the MLwiN software to identify risk factors associated with myopia. First, a univariate analysis was performed to evaluate potential associations. Then, multivariate logistic regression modeling was performed to analyze all statistically significant factors found in the univariate analysis. The odds ratio (OR) and corresponding 95% confidence interval (CI) were calculated to identify myopia risk factors. In the model, OR > 1.0 and *P* <  0.05 indicated that a parameter was a risk factor, while OR < 1.0 and *P* <  0.05 indicated that a parameter was a protective factor. All statistical tests were two-sided, and a value of *P* < 0.05 was considered statistically significant.

## Results

### Study population

As shown in Table 1, of the 997 children who completed the eye examinations and questionnaires, 52% were boys and 42.9% studied in rural schools. The mean (standard deviation) VA of the right eye was 4.83 ± 0.31, and that of the left eye was 4.84 ± 0.30.

**Table 1:** Demographic factors associated with myopia in children

Variables	Total, n (%)	No myopia, n (%)	Myopia, n (%)	*P* value
Total	997 (100)	659 (66.1)	338 (33.9)	
School region				
Rural	428 (42.9)	299 (69.9)	129 (30.1)	0.030
Urban	569 (57.1)	360 (63.3)	209 (36.7)	
Gender				
Male	523 (52.5)	366 (70.0)	157 (30.0)	0.007
Female	474 (47.5)	293 (61.8)	181 (38.2)	
Age (years)				
7	141 (14.1)	127 (90.1)	14 (9.9)	< 0.001
8	135 (13.5)	122 (90.4)	13 (9.6)	
9	153 (15.3)	115 (75.2)	38 (24.8)	
10	179 (18.0)	102 (57.0)	72 (40.2)	
11	195 (19.6)	92 (47.2)	103 (52.8)	
12	172 (17.3)	88 (51.2)	84 (48.8)	
13	22 (2.2)	8 (36.4)	14 (63.6)	

### Refractive error

Refractive error varied with grade level and sex in right eyes: mean SE was 0.15 D in grade 1 boys and 0.08 D in girls. Mean SEs were − 0.04 D, − 0.21 D, − 0.61 D, − 1.15 D, and − 1.05 D for boys from grades 2 to 6, respectively, while they were 0.08D, − 0.31D, − 0.89D, − 1.47 D, and − 1.55 D for girls in the same grades, respectively. In the left eyes, mean SEs were 0.27 D, 0.08 D, − 0.09 D, − 0.52 D, − 0.93 D, and − 0.93 D for boys from grades 1 to 6, respectively, while they were 0.19 D, 0.15 D, − 0.25 D, − 0.69 D, − 1.34 D, and − 1.38 D for girls in the same grades, respectively.

Refractive error varied with age and sex in right eyes: mean SEs were 0.14 D, 0.04 D, − 0.26 D, − 0.51 D, − 1.22 D, − 0.97 D, and − 1.15 D for boys aged 7 to 13 years, respectively, while they were 0.17 D, 0.01 D, − 0.28 D, − 0.97 D, − 1.54 D, − 1.43 D, and − 1.48 D for girls of the same ages, respectively. In the left eyes, mean SEs were 0.28 D, 0.11 D, − 0.09 D, − 0.44 D, − 0.99 D, − 0.94 D, and − 0.50 D for boys aged 7 to 13 years, respectively, while they were 0.27 D, − 0.03 D, − 0.10 D, − 0.81 D, − 1.34 D, − 1.29 D, and − 1.23 D for girls of the same ages, respectively.

### Prevalence of myopia

The overall prevalence of myopia was 33.9% in our study. When stratified according to myopia categories, low myopia had the highest prevalence, followed by moderate and high myopia (Table 2). Moreover, myopia prevalence significantly increased with increasing grade level (χ^2^ trend test = 127.63, *P* < 0.001), ranging from 11.0% in grade 1 to 51.9% in grade 6. Likewise, the prevalence of myopia significantly increased with age (χ^2^ trend test = 124.62, *P* < 0.001), ranging from 9.9% at age 7 to 63.6% at age 13. Moreover, the prevalence in girls was significantly higher than that in boys (38.2% vs. 30.0%, χ^2^ = 7.40, *P* = 0.007). Compared with children studying in rural schools, the prevalence of myopia was higher among children studying in urban schools (χ^2^ = 4.74, *P* = 0.03).

**Table 2:** Prevalence rate and categories of myopia

Variables	Myopia (%; 95% CI)	Myopia categories n (%)		
		Low	Moderate	High
Total	33.9; 31.0–36.8	258 (76.3)	73 (21.6)	7 (2.1)
School region				
Rural	30.1; 27.2–33.1	104 (80.6)	23 (17.8)	2 (1.6)
Urban	36.7; 33.8–39.7	154 (73.7)	50 (23.9)	5 (2.4)
Gender				
Male	30.0; 27.1–33.0	120 (76.4)	35 (22.3)	2 (1.3)
Female	38.2; 35.2–41.1	138 (76.2)	38 (21.0)	5 (2.8)
Age (years)				
7	9.9; 5.0–14.9	14 (100)	0 (0)	0 (0)
8	9.6; 4.7–14.6	12 (92.3)	1 (7.7)	0 (0)
9	24.8; 18.0–31.7	30 (78.9)	6 (15.8)	2 (5.3)
10	40.2; 33.0–47.4	58 (80.6)	13 (18.1)	1 (1.3)
11	52.8; 45.8–59.8	70 (68.0)	31 (30.1)	2 (1.9)
12	48.8; 41.4–56.3	62 (73.8)	20 (23.8)	2 (2.4)
13	63.6; 43.5–83.7	12 (85.7)	2 (14.3)	0 (0)

*CI* confidence interval

### Risk factors identified by multilevel logistic model

The data had a two-level hierarchical structure with 997 children nested within 24 classes. The level 1 units were individual children and the level 2 units were classes. First, a two-level null model without explanatory variables was fitted. The predictive or penalized quasi-likelihood was implemented in MLwiN to generate discrete response multilevel models. The linearization method, based on a Taylor series expansion, was performed to transform a discrete response model to a continuous response model; the model was then estimated using iterative generalized least squares [17].

Null model:

$$ \log \left({\pi}_{ij}\right)={\beta}_{0j} $$

$$ {\beta}_{0j}=-0.913(0.217)+{u}_{0j} $$

$$ {u}_{0j}\sim N\left(0,{\Omega}_u\right):{\Omega}_u=\left[0.960(0.322)\right] $$

In the null model, the intercept for class j was −0.913 (standard error = 0.217) + *u*_0*j*_, where the variance of *u*_0*j*_ was estimated as 0.960 (standard error = 0.322). The Wald chi-squared test statistic was 8.86 with a *P*-value of 0.003. Therefore, we concluded that there were significant differences between classes. We fitted a two-level random intercept model to analyze potential factors associated with myopia because the data appeared clustered at the class level.

**Fig. 1:**
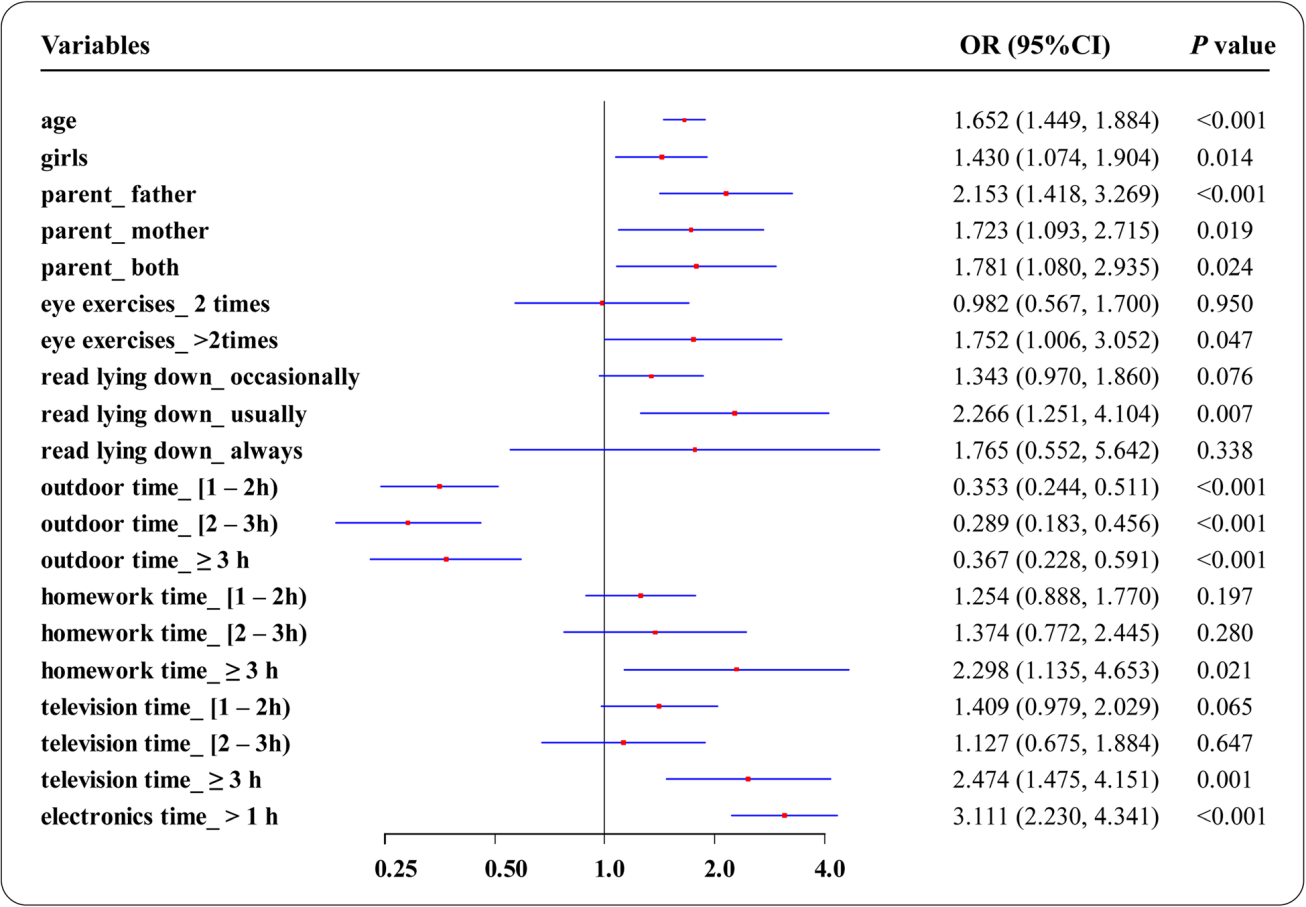
Associations between potential factors and myopia in univariate analysis. Myopia was significantly associated with age, sex, parental myopia, performing eye exercises, reading while lying down, less time outdoors, more time for homework, more time watching television, and more time playing with electronics; playing with electronics had the highest odds ratio of 3.111 (95% confidence interval: 2.230–4.341)

In the univariate analysis, the prevalence of myopia was significantly associated with increasing age (*P* < 0.001), female sex (*P* = 0.014), performing more eye exercises (*P* = 0.047), paternal myopia (*P* < 0.001), maternal myopia (*P* = 0.019), and reading while lying down (*P* = 0.007) as well as spending less time outdoors (*P* < 0.001), more time on homework (*P* = 0.021), more time watching television (*P* = 0.001), and more time playing with electronics (*P* < 0.001). The prevalence of myopia was not significantly associated with region, class recess location, sleeping duration, cramming for study sessions, computer usage, reading while in a dark environment, or reading distance (Fig. 1).

Full model:

$$ \log \left({\pi}_{ij}\right)={\beta}_{0j}+0.473(0.075)\mathrm{age}\_{1}_{ij}+0.371(0.159)\mathrm{gender}\_{1}_{ij}+0.756(0.223)\mathrm{parent}\_{1}_{ij}+0.621(0.244)\mathrm{parent}\_{2}_{ij}+0.447(0.278)\mathrm{parent}\_{3}_{ij}-0.980(0.195)\mathrm{outdoor}\_{1}_{ij}-1.335(0.245)\mathrm{outdoor}\_{2}_{ij}-1.101(0.258)\mathrm{outdoor}\_{3}_{ij}+0.325(0.182)\mathrm{homework}\_{1}_{ij}+0.370(0.320)\mathrm{homework}\_{2}_{ij}+0.805(0.390)\mathrm{homework}\_{3}_{ij}+0.386(0.198)\mathrm{television}\_{1}_{ij}+0.027(0.294)\mathrm{television}\_{2}_{ij}+0.745(0.287)\mathrm{television}\_{3}_{ij}+1.093(0.182)\mathrm{electronics}\_{1}_{ij} $$

$$ {\beta}_{0j}=-5.769(0.760)+{u}_{0j} $$

$$ {u}_{0j}\sim N\left(0,{\Omega}_u\right):{\Omega}_u=\left[0.192(0.101)\right] $$

$$ Var\left({myopia}_{ij}|{\pi}_{ij}\right)={\pi}_{ij}\left(1-{\pi}_{ij}\right)/{n}_{ij} $$

The definitions of the variables in the two-level random intercept model were shown in supplementary Table 1. In the multivariate analysis based on the full model, the presence of myopia was taken as the dependent parameter and all statistically significant factors in the univariate analysis were taken as independent variables. The analysis revealed that myopia was associated with age; female sex; paternal and maternal myopia; less time spent outdoors; more time spent on homework, watching television, and playing with electronics. The joint chi-squared test statistic for the full model was 169.673 (*P* < 0.001). The between-class variance decreased from 0.960 to 0.192; thus, some of the variation in myopia between classes was explained by the differences in age, sex, parental myopia, and time spent outdoors.

**Fig. 2:**
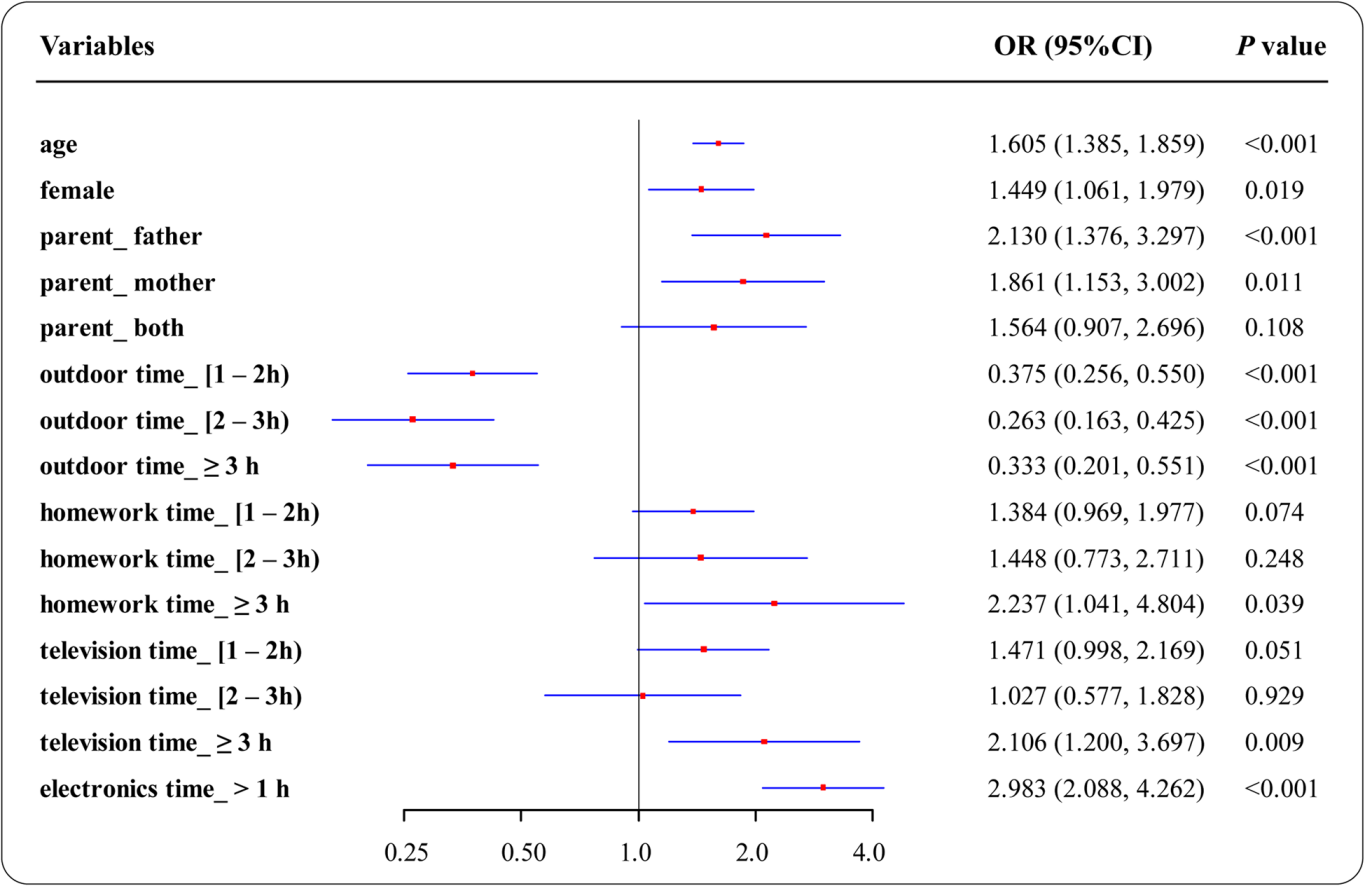
Associations between potential factors and myopia in multivariate analysis. Myopia was significantly associated with age, sex, parental myopia, less time outdoors, more time for homework, more time watching television, and more time playing with electronics; playing with electronics had the highest odds ratio of 2.983 (95% confidence interval: 2.088–4.262)

The results of multivariate analysis demonstrated that as children became older, their risk of myopia increased (OR = 1.605, 95% CI = 1.385–1.859) (Fig. 2). The results also showed that the prevalence of myopia was higher in girls than in boys (*P* = 0.019); girls had a 1.449-fold (95% CI = 1.060–1.979) higher risk of myopia than boys. Furthermore, the children with paternal myopia (OR = 2.130, 95% CI = 1.376–3.297) or maternal myopia (OR = 1.861, 95% CI =1.153–3.002) had a higher risk of myopia than those without myopic parents. In analysis of outdoor and indoor activities, children who spent more than 1 h daily outdoors had a lower risk of myopia (> 1 h, OR = 0.375, 95% CI = 0.256–0.550; > 2 h, OR = 0.263, 95% CI = 0.163–0.425; > 3 h, OR = 0.333, 95% CI = 0.201–0.551). Children who spent more than 3 h daily on homework, watched television more than 3 h daily or played with electronics more than 1 h daily all had a higher risk of myopia; the respective ORs were 2.237 (95% CI = 1.041–4.804), 2.106 (95% CI = 1.200–3.697), and 2.983 (95% CI = 2.088–4.262). The variance inflation factor (VIF) is used to quantify multicollinearity severity in statistical analyses. It provides an index that measures the extent of increase in the variance of an estimated regression coefficient because of collinearity. VIF values ranging from 0 to 10 are considered not to indicate multicollinearity. In the multivariate model, the VIFs of age, sex, parental myopia status, outdoor time, homework time, television time, and electronics time were 1.034, 1.033, 1.046, 1.108, 1.066, 1.160, 1.051, respectively. Hence, there was no multicollinearity in our multivariate analysis.

## Discussion

To the best of our knowledge, the present study is the first investigation of the prevalence of myopia and its associated risk factors among primary school children in Chongqing. The overall prevalence of myopia among primary school children in Chongqing was 33.9%, which was higher than the prevalence reported in several other countries: 3.3% in children aged 6–7 years and 19.9% in children aged 12–13 years in Ireland [18], as well as in Malaysia [4] and Korea [5]. Furthermore, the prevalence of myopia in Chongqing was higher than that in some areas of China, as reports indicated a prevalence of 2.4% in grade 1 students in Mojiang [19], 23.1% in children aged 6–18 years in Handan [20], and 26.4% in adults in Yunnan [21]; these data suggest that myopia is a serious public health problem in Chongqing along with other areas of China.

In most prior studies [1, 9, 11], ordinary logistic regression was performed to identify myopia risk factors; however, the intra-cluster dependency of hierarchically structured data can violate the assumption of independence in ordinary logistic regression, resulting in biased estimates. Hence, a multilevel model was used in the present study to assess hierarchically structured data. Multilevel modeling provides better statistical models that match real-world data, because it can address parameters that vary at more than one level, examine cross-level effects, and simultaneously decompose the variances of the variables into within-group and between-group components [13, 22]. The two-level logistic model used in this study separated the effects of individual myopia from those of the average myopia in a class. Although performing eye exercises and reading while lying down were significantly associated with myopia in univariate analysis; they were not significantly associated with myopia in the multivariate analysis. Our cross-sectional study using multilevel modeling revealed that myopia was associated with increased age, female sex, parental myopia, shorter time outdoors, and longer time indoors.

The higher prevalence of myopia among children in urban schools, compared with those in rural schools, was identified by the chi-squared test; this was not found in multilevel model, because the influence of school region, accepted as an independent variable in the two-level model, had already been addressed in the two-level model. Similar to a previous study [23], the higher prevalence of myopia among children in urban schools might result from more time spend on indoor activities than outdoor activities. We also found that the prevalence of myopia in girls was higher than that in boys, which was consistent with the findings in other Chinese locations, such as Guangzhou [15] and Xichang [24]. Previous studies indicated that older children had a higher risk of myopia [2, 15, 25], which was further confirmed by our study.

As in other countries [6, 26] and other large cities in China [15, 27], we found that parental myopia, typically regarded as a hereditary risk factor for myopia in different age and ethnic groups, was a significant risk factor associated with myopia in Chongqing. In a prospective cohort study, Saw et al. confirmed that parental myopia remained a significant risk factor for myopia, regardless of the definitions of incident myopia [26]. In addition, Guo et al. reported a significant positive relationship between the incidence of myopia and parental myopia in a longitudinal cohort of children in Beijing [10]. However, we did not find a dose-dependent pattern between myopia prevalence in children and the myopic status of their parents [12]; this may have been because the participants in their study were different from those in our study, and because the dose-dependent association may only play a role in older children who have already been exposed to environmental risk factors.

In addition to this genetic factor, environmental factors contribute significantly to the prevalence of myopia [28]. As in previous cross-sectional studies [3, 24, 27, 29], our study also demonstrated that more time involved in outdoor activities had protective effects on eye health. The prevalence of myopia in children of Chinese ethnicity was significantly higher than that in children in Singapore (29.1%) and in Sydney (3.3%); notably, children in Sydney spent substantially more time for outdoor activities [29]. Also, prospective cohort studies demonstrated that shorter time outdoors was a statistical risk factor for myopia. Guggenheim et al. reported that both time spent outdoors and physical activity were associated with incident myopia, and that time spent outdoors had a greater effect [30]. Guo et al. [10] found that, in univariate analysis, the incidence of myopia was significantly associated with a short time outdoors, and long times indoors focused on studying; however, multivariate analysis revealed that, only a short time outdoors was significantly associated with a higher incidence of myopia. In addition to the evidence provided by these cross-sectional and prospective cohort studies, a randomized clinical trial in China showed that outdoor activity can reduce the incidence rate of myopia [31]. Furthermore, the association between outdoor activity and myopia has been demonstrated in different age and ethnic groups [11, 32, 33].

Increased time spent on indoor activities, including homework, watching television, and playing with electronic devices, increased the risk of myopia in the present study; this finding was similar to that in a study conducted in Beijing [27]. In addition, Harrington et al. [9] found that there was a dose-dependent relationship between myopic prevalence of schoolchildren and the leisure time they spent in reading/writing. Contrary to the reports of previous studies that near work was a significant risk factor for myopia [7, 8, 34], the average reading distance was not associated with myopia in the present study; however, a related practice, reading while lying down was associated with myopia in our univariate analysis. Notably, this was not a risk factor in our multivariate analysis. Interestingly, the results of the two-level model showed that playing with electronics more than 1 h daily was a significant risk factor for myopia with the highest OR value (OR = 3.111, 95% CI = 2.230–4.341), indicating that this activity had the greatest effect on the risk of myopia; this finding was not reported in other studies conducted in China [2, 15]. Considering the above risk factors for myopia, public intervention measures related to increasing the time outdoors should be implemented to reduce the incidence of myopia.

A potential limitation in this study should be mentioned. When the prevalence rate is less than 10%, the OR value is similar to the prevalence rate ratio (PRR) value. However, the prevalence rate of myopia was 33.9% in our study, so the OR is different from the PRR and substantially overestimates the relative risk of myopia.

## Conclusions

In summary, the overall prevalence of myopia was relatively high in the present study, suggesting that myopia is a serious public health problem among school children in Chongqing. Myopia was significantly positively associated with female sex, increased age, parental myopia, shorter time outdoors, and longer time indoors. Importantly, playing with electronics more than 1 h daily had the greatest effect on risk of myopia.

## Supplementary information

**Additional file 1: Table S1.** Definitions of variables in two-level random intercept model.

## Data Availability

The datasets used and/or analyzed during this study are available from the corresponding author upon reasonable request.

## Abbreviations

CI: Confidence interval
D: Diopters
OR: Odds ratio
SE: Spherical equivalent refraction
VA: Visual acuity
