# Supplementary material for: Prevalence of myopia and associated risk factors among primary students in Chongqing: multilevel modeling
Source: BMC Ophthalmol. 2020 Apr 15;20:146. doi: 10.1186/s12886-020-01410-3 (PMC7161106; doi:10.1186/s12886-020-01410-3)
Supplement: Supplementary file 1 — Additional file 1: Table S1. Definitions of variables in two-level random intercept model. [file 12886_2020_1410_MOESM1_ESM.docx]

**Additional file 1: Supplementary table 1**. Definitions of variables in two-level random intercept model.

| Variables | Name | Description |
| --- | --- | --- |
| Dependent variable |  |  |
| Y | Myopia | No = 0, Yes = 1 |
| Independent variables |  |  |
| X_1_ | School region | Rural = 0, Urban = 1 |
| X_2_ | Sex | Male = 0, Female = 1 |
| X_3_ | Age | Age of children at time of survey |
| X_4_ | Parental myopia statuses | No = 0, Father = 1, Mother = 2, Both = 3 |
| X_5_ | Performing eye exercises | Once = 0, Twice = 1, At least three times = 2 |
| X_6_ | Class recess | Indoor = 0, Outdoor = 1 |
| X_7_ | Daily hours of sleep | > 9 h = 0, ≤ 1 h = 1 |
| X_8_ | Daily hours of outdoors | < 1 h = 0, [1 – 2h) = 1, [2 – 3h) = 2, ≥ 3 h = 3 |
| X_9_ | Daily hours of doing homework | < 1 h = 0, [1 – 2h) = 1, [2 – 3h) = 2, ≥ 3 h = 3 |
| X_10_ | Weekly hours of attending cram school | < 1 h = 0, [1 – 2h) = 1, [2 – 3h) = 2, ≥ 3 h = 3 |
| X_11_ | Daily hours of watching television | < 1 h = 0, [1 – 2h) = 1, [2 – 3h) = 2, ≥ 3 h = 3 |
| X_12_ | Daily hours of using computer | < 1 h = 0, [1 – 2h) = 1, [2 – 3h) = 2, ≥ 3 h = 3 |
| X_13_ | Daily hours of playing electronics | ≤ 1 h = 0, > 1 h = 1 |
| X_14_ | Reading while in dark environment | No = 0, Occasionally = 1, Usually = 2, Always = 3 |
| X_15_ | Reading while lying down | No = 0, Occasionally = 1, Usually = 2, Always = 3 |
| X_16_ | Reading distance ≤ 33cm | No = 0, Occasionally = 1, Usually = 2, Always = 3 |
| Level 2 | Class | Identifying code for each class |
| Level 1 | Child | Identifying code for each child |

h, hour.
